# Influence of CReatine supplementation on mUScle mass and strength after stroke (ICaRUS Stroke Trial): study protocol for a randomized controlled trial

**DOI:** 10.1186/s13063-023-07248-6

**Published:** 2023-03-22

**Authors:** Juli Thomaz de Souza, Marcos F. Minicucci, Natália C. Ferreira, Bertha F. Polegato, Marina Politi Okoshi, Gabriel P. Modolo, Bethan E. Phillips, Philip J. Atherton, Kenneth Smith, Daniel Wilkinson, Adam Gordon, Suzana E. Tanni, Vladimir Eliodoro Costa, Maria Fernanda P. Fernandes, Silméia G. Zanati Bazan, Leonardo A. M. Zornoff, Rodrigo Bazan, Sérgio A. Rupp de Paiva, Paula Schmidt Azevedo

**Affiliations:** 1 Department of Internal Medicine, São Paulo State University (UNESP) Medical School, Botucatu, SP Brazil; 2 Department of Neurology, Psychology and Psychiatry, São Paulo State University (UNESP) Medical School, Botucatu, SP Brazil; 3 Centre for Musculoskeletal Ageing Research & NIHR Nottingham BRC Clinical, Metabolic and Molecular Physiology, University of Nottingham, Royal Derby Hospital Centre Uttoxeter Road, Derby, England; 4 Stable Isotopes Center, Institute of Biosciences, São Paulo State University (UNESP), Botucatu, SP Brazil

**Keywords:** Stroke, Creatine, Skeletal muscle, Sarcopenia

## Abstract

**Background:**

Stroke is a leading cause of mortality and disability, and its sequelae are associated with inadequate food intake which can lead to sarcopenia. The aim of this study is to verify the effectiveness of creatine supplementation on functional capacity, strength, and changes in muscle mass during hospitalization for stroke compared to usual care. An exploratory subanalysis will be performed to assess the inflammatory profiles of all participants, in addition to a follow-up 90 days after stroke, to verify functional capacity, muscle strength, mortality, and quality of life.

**Methods:**

Randomized, double-blind, unicenter, parallel-group trial including individuals with ischemic stroke in the acute phase. The duration of the trial for the individual subject will be approximately 90 days, and each subject will attend a maximum of three visits. Clinical, biochemical, anthropometric, body composition, muscle strength, functional capacity, degree of dependence, and quality of life assessments will be performed. Thirty participants will be divided into two groups: intervention (patients will intake one sachet containing 10g of creatine twice a day) and control (patients will intake one sachet containing 10g of placebo [maltodextrin] twice a day). Both groups will receive supplementation with powdered milk protein serum isolate to achieve the goal of 1.5g of protein/kg of body weight/day and daily physiotherapy according to the current rehabilitation guidelines for patients with stroke. Supplementation will be offered during the 7-day hospitalization. The primary outcomes will be functional capacity, strength, and changes in muscle mass after the intervention as assessed by the Modified Rankin Scale, Timed Up and Go test, handgrip strength, 30-s chair stand test, muscle ultrasonography, electrical bioimpedance, and identification of muscle degradation markers by D3-methylhistidine. Follow-up will be performed 90 days after stroke to verify functional capacity, muscle strength, mortality, and quality of life.

**Discussion:**

The older population has specific nutrient needs, especially for muscle mass and function maintenance. Considering that stroke is a potentially disabling event that can lead the affected individual to present with numerous sequelae, it is crucial to study the mechanisms of muscle mass loss and understand how adequate supplementation can help these patients to better recover.

**Trial registration:**

The Brazilian Clinical Trials Registry (ReBEC) RBR-9q7gg4. Registered on 21 January 2019.

## Background

Stroke is an important public health problem and is one of the leading causes of death in Brazil [1]. Stroke is the main cause of disability in the adult population, and approximately two-thirds of patients experience an incomplete recovery. Malnutrition on admission, reduced level of consciousness, dysphagia, and enteral tube feeding use may impair the nutritional status of these individuals, who generally become more susceptible to involuntary weight loss at this stage [2, 3].

Studies on the acute phase of stroke have shown that patients who were malnourished at the time of ictus had more complications and higher mortality rates than patients with adequate weight, overweight, or obesity [4].

After stroke, appetite loss, dysphagia, depression, changes in mobility, and functional dependence can lead to inadequate food intake, which is associated with neurological damage. Moreover, bed rest compromises nutritional status, resulting in a negative effect on physical and cognitive abilities [5, 6].

A previous study on patients in the acute phase of stroke demonstrated that individuals with some degree of dysphagia at the time of hospital discharge had higher mortality rates, regardless of stroke severity and other clinical factors, which may indicate the importance of adequate nutritional management during hospitalization for stroke and monitoring after hospital discharge for patients with food intake difficulties [7].

Malnutrition has been associated with inflammation and metabolic stress, which are proportional to injury severity, and increased protein catabolism, hypermetabolism, and insulin resistance [8].

Sarcopenia is associated with aging and is commonly observed in patients after stroke. Sarcopenia is considered primary when the patient has skeletal muscle mass decline, reduced water content, and increased body fat, or secondary to chronic or disabling diseases such as stroke [8, 9].

Unlike age-related sarcopenia, stroke-related sarcopenia has specific characteristics, such as rapid muscle mass decline and structural changes. The type of stroke and location and size of the brain injury are important factors affecting the differences in physical and functional performance, depending on the side affected by stroke, which is independent of age. Moreover, catabolic signals from brain damage can result in an imbalance in the neurovegetative state. Therefore, there is a complex metabolic and systemic change in individuals with stroke that can lead to weight loss and reduced anabolism, worsening the prognosis [10]. Muscle changes occur within 4 h after stroke, and over 7 days, patients can develop muscle weakness on both the affected and contralateral sides of the body [11].

Daily intake of 1.2 to 1.5 g of protein per kg of body weight is recommended along with exercise for sarcopenia prevention in older adults, as this population is more susceptible to inadequate protein intake for several aging-related reasons leading to increased proteolysis and anabolic resistance [12, 13]. However, limited data are available regarding the prevention of sarcopenia or malnutrition in the acute phase after stroke.

Some nutrients and amino acids such as creatine appear to be potential compounds capable of promoting hypertrophy and improving muscle function. Creatine supplementation has been proven effective in improving muscle mass in healthy older individuals, even when only taken for a few days; in pathological conditions, these positive results are supported by studies in several populations [14–17].

A combination of resistance exercise and adequate protein intake is recommended to maintain healthy skeletal muscles. However, there is no recommendation for this type of multimodal therapy in older people after stroke. Evidence is scarce regarding the influence of protein supplementation and specific amino acids associated with physical activity on the functional capacity and quality of life among this population.

The aim of the study is to verify the effectiveness of creatine supplementation on functional capacity, strength, and changes in muscle mass during hospitalization for stroke compared to usual care. A descriptive and comparative evaluation between groups in order to verify the inflammatory and potential serum biomarkers behavior during hospitalization will be performed with all participants randomized. A follow-up 90 days after stroke to verify functional capacity, muscle strength, mortality, and quality of life will be carried out.

## Methods

### Trial design

**Fig. 1:**
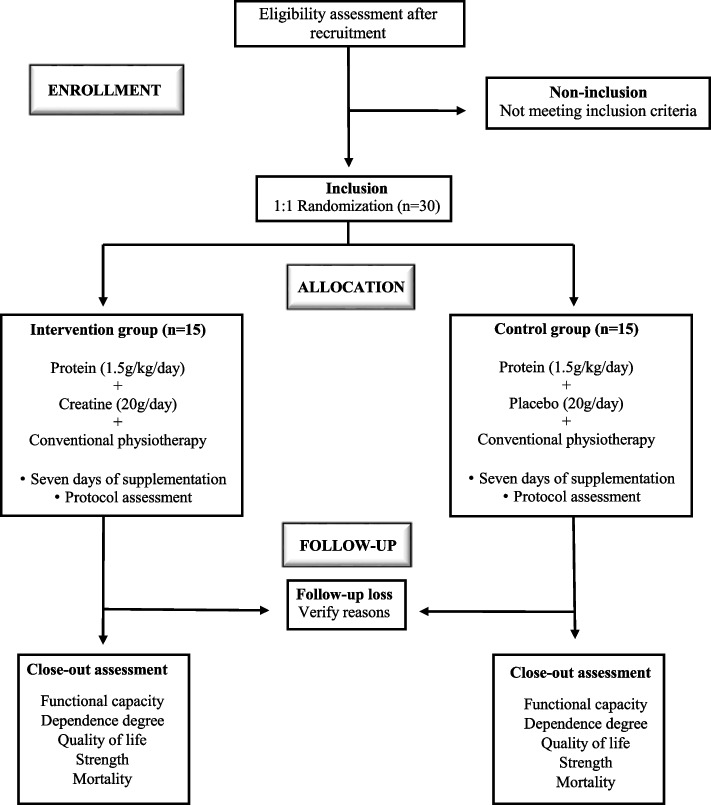
Flow diagram of the ICaRUS Stroke Trial

It will be a (randomization 1:1 to the intervention or control groups) double-blind (participants and researchers responsible for administering the supplement and carrying out the protocol assessments), unicenter, parallel-group trial including individuals with ischemic stroke in the acute phase to verify the superiority of the intervention compared to the control group. The duration of the trial for the individual subject will be approximately 90 days, and each subject will attend a maximum of three visits. The protocol was written according to the 2013 Standard Protocol Items: Recommendations for Interventional Trials (SPIRIT) guidelines. A flow diagram of the study is presented in Fig. 1.

### Participants and eligibility criteria

Men and women aged 60 years and older who were diagnosed with ischemic stroke without previous disability (Modified Rankin Scale [mRs] – [mRs≤2]) and agree to sign the informed consent form will be recruited within 24 h of stroke.

Patients with any of the following will not be included: hemodynamic instability, mechanical ventilation, pacemakers and metallic prostheses, previous kidney disease, creatinine clearance ≤30 ml/min/1.73 m^2^, history of disabsorptive gastrointestinal surgery, parenteral nutrition, allergies or intolerance to any component of the study products, and patients who decline to participate.

The study will be conducted at the Stroke Unit of the Clinical Hospital of Botucatu Medical School, Brazil, and the follow-up visits at the Neurovascular Disease Outpatient Clinic of the same institution.

### Interventions

Creatine supplementation will be administered in the intervention group to avoid the muscle loss that is common in patients after stroke. The control group will receive maltodextrin, a complex carbohydrate with no direct effect on muscle loss. All participants will receive the same amount of protein per kg of body weight so that the groups are homogeneous in the consumption of this nutrient. The intervention will proceed as follows:

Intervention group (15 participants)—Will be administered one sachet containing 10 g of creatine twice a day.

Control group (15 participants)—Will be administered one sachet containing 10 g of placebo (maltodextrin) twice a day.

All sachets will be the same color and size. All patients will receive assisted supplementation with powdered milk protein serum isolate, if necessary, to achieve a daily intake of 1.5 g/kg of body weight and standard physiotherapy care. Supplementation will be offered during the 7-day hospitalization.

Rehabilitation will start after the first 24 h of the ictus, and the intensity will be proportional to the benefit and tolerance of each individual. Standard physiotherapy will be performed according to the current rehabilitation guidelines for stroke patients and will consist of contracture prevention, limb and trunk strengthening, gait training, and independence [18, 19].

All individuals will be instructed at the time of hospital discharge on healthy eating habits, and adequate fractioning, and provided incentives for eating fruits, vegetables, and whole grains, and maintaining water intake in accordance with the Dietary Guidelines for the Brazilian Population by the Ministry of Health [20].

Participants who are intolerant to the supplements or who withdraw their consent will be excluded from the study. All patients will be followed up daily and encouraged to consume all meals and study products. If necessary, substitutions will be made to the patient’s diet with similar foods to promote supplementation acceptance during the intervention period.

### Outcomes

The primary outcomes will be functional capacity, strength, and changes in muscle mass after the intervention. The evaluations will be conducted in the first 24 hours after stroke and on the 7^th^ day of hospitalization. The patients will be assessed for the following:

1. Functional capacity according to the mRs and Timed Up and Go test scores.
2. Muscle strength according to handgrip strength and lower limb strength according to the National Institutes of Health Stroke Scale (NIHSS) score.
3. Muscle mass by muscle ultrasonography, electrical bioimpedance, and muscle degeneration markers by D3-methylhistidine.

A descriptive and comparative evaluation between groups in order to verify the inflammatory and potential serum biomarkers behavior during hospitalization will be performed with all participants randomized.

### Follow-up evaluations

A follow-up 90 days after stroke will be conducted to verify functional capacity, muscle strength, mortality, and quality of life. The following will be assessed in each patient:

1. Functional capacity according to the mRs and Timed Up and Go test scores.
2. Muscle strength based on handgrip strength and lower limb strength according to NIHSS score and the 30-s chair stand test score.
3. Patient perception of quality of life according to The European Quality of life Scale Five Dimension (EuroQol-5D) score.

### Procedures (template)

All patients with ischemic stroke confirmed by imaging examinations who meet the study inclusion criteria will be invited to participate. After signing the informed consent form, the patients will be randomized to the intervention or control groups. All patients will undergo clinical and neurological evaluations conducted by the medical team using the NIHSS score, which is used to determine treatment and predict prognosis; Bamford classification according to brain injury location; and the Alberta Stroke Program Early CT Score (ASPECTS), which verifies the extent of the injury [1, 21].

**Fig. 2:**
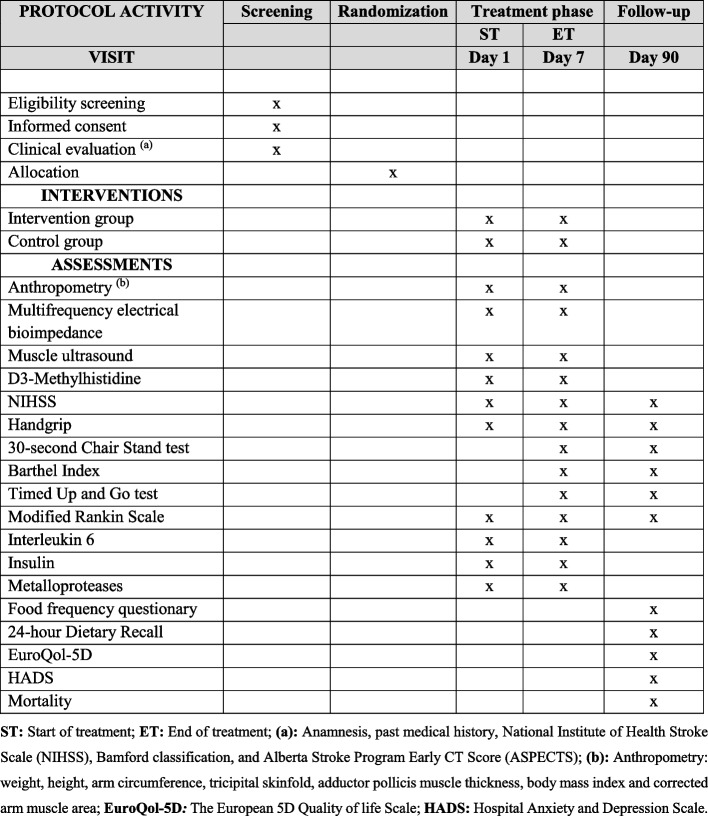
ICaRUS Stroke Trial template of recommended content for the schedule of screening, interventions, and assessments

The template for the recommended schedule of enrollment, interventions, and assessments is presented in Fig. 2. To verify the occurrence of mortality, we will utilize death certificates from medical records.

### Sample size

We reviewed a study that analyzed the effects of creatine supplementation in 18 healthy older people, in which 10 patients were allocated to the creatine group and 8 to the control group. The authors observed an improvement in muscle strength, weight, and fat-free mass in the group that received supplementation [22]. In addition, a meta-analysis based on this previous study included approximately 600 patients from 60 studies, with an average of 10 patients in each arm per study [16]. Considering that this is a study on creatine supplementation in patients after stroke, we decided to include 50% more in the convenience sample, with a total of 15 patients in each group.

### Recruitment

Daily monitoring of admissions to the Stroke Unit of the Clinical Hospital of Botucatu Medical School, Brazil, will be carried out to refer individuals for screening and checking of inclusion criteria.

### Randomization and blinding

For all subjects, the screening visit and the enrollment visit were performed on the same day. Subjects were randomized 1:1 to the intervention or control groups. The randomization was performed using a cell phone application, which creates a random control and intervention sequence, and was performed in 10 out of 10 participants. No stratification factor was used.

To maintain the blindness of the study, randomization will be performed by a person uninvolved in data collection and data analysis. The participant, the professional responsible for administering the supplements, and the person responsible for conducting the protocol assessments will be blinded to the intervention. The professional in charge of the statistical analysis of the data will not be blinded to the intervention.

Breaking the blinding will be allowed if the participant has a serious adverse effect related to the supplementation, such as an allergic reaction. In this case, the principal investigator will be responsible for breaking the blinding and for informing the hospital’s medical staff about which supplement the participant was consuming.

### Data collection, management, and analysis

All collected data will be de-identified and stored in a safe place to protect the confidentiality of each participant. After 5 years from the end of the research, the data will be destroyed. Subjects in both groups will be evaluated in the first 24 h after the stroke, after 7-day hospitalization, and 90 days after stroke. All individuals who participate in the study will be instructed to complete the protocol during the 7 days of hospitalization in the stroke unit. The research team will carry out daily visits during the 7 days of hospitalization to guarantee the participant’s permanence in the study. After hospital discharge, the team will contact the participant by telephone to ensure the follow-up visit 90 days after the stroke. In case of loss of contact, the researchers will count on the social service of the hospital to carry out an active search for the participant.

All data will be double-checked by 2 team researchers to promote data quality (e.g., double data entry; range checks for data values).

### Baseline assessments

#### Anthropometric evaluation

Anthropometric evaluation will be performed by the same evaluator and will include weight, height, arm circumference, tricipital skinfold, and adductor pollicis muscle thickness. Subsequently, the body mass index will be calculated according to the Quetelet equation and the arm muscle area will be corrected using the formula proposed by Frisancho [23, 24].

For anthropometric measurements, the following equipment will be used: weight scale (Relaxmedic Your Way), portable stadiometer (InLab), inelastic and inextensible measuring tape with values in centimeters, and an adipometer (Lange®; Cambridge Scientific Industries, Watertown, MA, USA). Anthropometric assessments will be performed on both the affected and unaffected sides of the body.

The thickness of the rectus femoris and biceps brachii muscles, both affected and unaffected by the stroke, will be measured with a BodyMetrix BX-2000 A mode ultrasound device (Intelametrix, Livermore, CA, USA) with a 2.5-MHz transducer. Each location will be measured two or three times, based on the software’s compliance with these measurements, and the average will be used to represent the final thickness [25].

#### Body composition assessment

Multifrequency electric bioimpedance will be performed with the SECA mBCA525 body composition analyzer, with the patients in a supine position, with their legs apart, hands open, palms down, and separated from the body. Eight tetrapolar electrodes will be connected to assess the impedance of the trunk, arms, and legs at six different frequencies (1, 5, 50, 250, 500, and 1000 kHz).

#### Muscle strength assessment

Handgrip strength will be measured using a hydraulic hand dynamometer (Saehan Hydraulic Hand Dynamometer®, Model SH5001, Saehan Corporation, Korea), which measures the force produced by an isometric contraction applied to the handles, and the value will be recorded in kilogram force. Three measurements will be performed and the maximal value achieved will be recorded with a 15-s rest interval between measurements on the affected and unaffected sides [26].

Lower limb strength will be assessed according to the 30-s chair stand test using a chair placed against a wall; the test begins with the patient sitting in the chair, with their back straight, feet aligned with the shoulders, and the arms crossed at the wrists and held against the chest. At the “go” signal, the patient will stand up straight and then return to the initial seated position. The number of correctly executed cycles within 30 s will be counted, and the incorrect cycles will be discarded [27].

The NIHSS item 6 score will be used to verify lower limb strength. The patient will be placed in a supine position with the evaluated leg extended to 30°, and we will assess whether the limb falls within 5 s. Each limb is tested in turn, beginning with the nonparetic leg. The examiner will only record the score as untestable in cases of amputation or joint fusion at the hip. This item is scored as follows: 0 = no drift, the leg is held in the 30-degree position for the full 5 s; 1 = drift, the leg falls before the end of the 5-s period but does not touch the bed; 2 = some effort against gravity, the leg falls to the bed before 5 s although there is some effort made against gravity; 3 = no effort against gravity, the leg falls to the bed immediately; 4 = no movement; and UN = untestable owing to amputation or joint fusion at the hip [28].

#### Functional capacity assessment

The mRs will be applied, with scores ranging from 0 to 6; the higher the value, the greater the degree of disability. Death is classified as a score of 6 [21].

The Timed Up and Go test will assess mobility and balance by measuring the time it takes the patient to get up from a chair, walk in a straight line for 3 m (at a comfortable and safe pace), turn around, walk back, and return to a seated position on the chair [29].

#### Dependence degree assessment

The Barthel Index will be used to measure functional independence and mobility in chronically ill patients. The scale consists of 15 items, with scores ranging from 0 to 100 (0–20 indicating total dependence; 21–60 severe dependence; 61–90 moderate dependence; 91–99 light dependence, and 100 independence) [21].

#### Quality of life assessment

The EuroQol-5D will be used to assess the individuals’ perceptions of quality of life through five domains of mobility, personal care, usual activities, pain/discomfort, and anxiety/depression; the higher the value, the worse the quality of life perception. At the end of the questionnaire, the patient must indicate their health status using an ordinal scale from 0 to 100; the closer to 0 the worse their health status and the closer to 100 the better their health status [17].

#### Assessment of anxiety and depression

The Hospital Anxiety and Depression Scale will be used to screen for anxiety and depression and assess the severity of mood disorders [30].

#### Biochemical dosages

In the first 24 h after stroke and on the 7th hospitalization day, the patient will have blood samples collected for the analysis of insulin and interleukin-6 levels by enzyme-linked immunoassay, in which the amount of protein in the extract will be determined using the Bradford method with the final concentration adjusted to 1 mg/ml. The activity of metalloproteases 2 and 9 will be obtained by zymography as described by Tyagi et al. [31].

#### Stable isotope tracer analysis

Compound 3-methylhistidine is a product of the breakdown of contractile proteins and is a marker of muscle degeneration. In the first 24 h after stroke, and on the 7th day of hospitalization, the patient will have blood samples collected for residual 3-methylhistidine evaluation. The patient will then receive 50 mL of a solution containing 10 mg of 99.1% pure D3-methylhistidine (Cambridge Isotope Laboratories, Andover, MA, USA). After 18 h, three 10-mL blood samples will be collected at 1-h intervals. Of 30 patients, 10 will be included in the analysis. The samples will be sent to the Centre for Musculoskeletal Aging Research & NIHR Nottingham BRC (Clinical, Metabolic, and Molecular Physiology research group, University of Nottingham, Royal Derby Hospital Centre, Derby, UK). The ratio between labeled and unlabeled 3-methylhistidine will be evaluated using liquid chromatography-mass spectrometry [32–34].

### Statistical analysis

The Full Analysis Set (FAS) will comprise all randomized subjects who have received at least one dose of creatine. The subjects will contribute to the analysis as randomized. The FAS will be used for efficacy analysis instead of intention-to-treat analysis.

The Per-Protocol (PP) population will comprise all subjects in the FAS who do not have any major protocol deviations (including but not limited to violation of inclusion and exclusion criteria), and who have at least good compliance as reported in the eCRF and for whom the primary endpoint can be derived. The PP will be used for the analysis of the primary endpoint.

The Safety Analysis Set will comprise all subjects who have received at least one dose of creatine or placebo. The subjects will contribute to the analysis as actually treated. The Safety Analysis Set will be used for the evaluation of safety endpoints and the primary endpoint.

Missing values will be treated mathematically by multiple imputation.

All data will be analyzed using Stata SE version 15.0 (StataCorp LLC., College Station, TX, USA).

#### Planned statistical methods

- Statistical Considerations Baseline is defined as the randomization visit and the end of the trial is defined as the final visit. Categorical data will be summarized by treatment group, using the number and percentages of subjects.
- For the calculation of percentages, the denominator will be the number of subjects in the analysis set.
- Continuous data will be presented using the number of subjects, mean, standard deviation, median, lower quartile, upper quartile, minimum, and maximum. Both the absolute values and the change from baseline will be presented. The treatment group and visit will present descriptive statistics for all endpoints (if applicable). All statistical tests will be carried out as two-sided and performed on a 5 % significance level. Estimated treatment differences and 95% CIs will be presented together with the corresponding *p*-value. All safety endpoints will be presented using descriptive statistics and no formal statistical tests will be applied.
- The evaluation of continuous variables of the characteristics between the groups will be analyzed by the *t*-test or Mann-Whitney.
- The assessment of variation of serum levels of biomarkers, muscle mass, and strength between groups and time will be done by the mixed regression models.
- Kaplan-Meier survival curves will be used to show the survival in the groups within 90 days. Considering day zero as the day of randomization and entering into the study, patients related the final date they had each outcome. This is appropriate as it takes into account what happened during the course of the study. *P*-values were obtained through proportional Cox regression models and a value of 5% was considered statistically significant.
- All adverse events will be summarized. The summaries will include the number of events, the number of subjects, and the proportion of subjects reporting these events and will be tabulated by system organ class.

#### Sensitivity analysis

The primary analysis will be repeated using the PP and safety analysis set. We will perform analysis using only patients with specific symptoms to each symptoms.

### Data monitoring

The study follows the schedule of the Research Ethics Committee of Sao Paulo State University with the submission of partial reports to assess the progress and conduct of the study. At the end of the study, the final report will be sent with all the results. As this is an intervention with a low risk of serious adverse events, with a supplement known to be safe at the doses administered in the study, interim analyzes are not foreseen before the end of the study.

Non-serious adverse events such as diarrhea, dyspepsia, and abdominal pain related to supplementation will be analyzed individually and discussed with the assisting medical team to assess what action will be taken. Serious adverse events related or not to the intervention, such as death, acute kidney injury, infection with prolonged hospitalization, and hospitalization for any cause will be reported to the institution’s Research Ethics Committee within 24 h of their occurrence. All adverse events will be summarized. The summaries will include the number of events, the number of subjects, and the proportion of subjects reporting these events.

Monthly team meetings are held with the principal investigator to align the protocol, verify the progress of the study, and ensure compliance with deadlines. Additionally, the study will have an external endpoint adjudication committee composed of three physicians independent from investigators and the sponsor to investigate adverse events.

## Discussion

This clinical trial encompasses the use of creatine in the acute phase of stroke. Indeed, research about therapeutic strategies to mitigate the muscle mass decline after stroke is urgent. In general, the researches are focused on assessment, instead of treatment.

The limitation of the study is the sample size. However, the studies involving nutritional interventions to old people are a big deal, because of compliance and preferences. In addition, the stroke may add an extra difficulty because of the chance of conscience level, dysphagia, and tube feeding. In fact, studies published in a systematic review involving creatine supplementation in different scenarios included an average of 10 patients per group, showing that creatine might exert benefits for muscle mass and function [16]. On the other hand, the study will be rigorously conducted inside a stroke unit, with intake control, standardized care, and monitoring.

In addition, the trial may bring information for next larger studies, especially regarding side effects, compliance, and future sample size calculation. ICaRUS Stroke Trial will not be conducted as a feasibility study because we intend to make an entirely statistical analysis and follow-up.

### Trial status

This trial is ongoing. Recruitment and intervention phase started in March 2019 and recruitment is expected to end in June 2023.

## Data Availability

The results will be published in scientific journals and specific conferences in the study area. Anonymized data may be made available for research purposes by contacting the corresponding author. Any additional information or data required to support the protocol may be obtained from the corresponding author by email.

## Abbreviations

SPIRIT: Standard Protocol Items: Recommendations for Interventional Trials
mRs: Modified Rankin Scale
EuroQol-5D: The European 5D Quality of life Scale
NIHSS: National Institute of Health Stroke Scale
ASPECTS: Alberta Stroke Program Early CT Score
